# Moderate salt treatment alleviates ultraviolet-B radiation caused impairment in poplar plants

**DOI:** 10.1038/srep32890

**Published:** 2016-09-06

**Authors:** Xuan Ma, Yong-Bin Ou, Yong-Feng Gao, Stanley Lutts, Tao-Tao Li, Yang Wang, Yong-Fu Chen, Yu-Fang Sun, Yin-An Yao

**Affiliations:** 1 School of Life Science and Engineering, Southwest University of Science and Technology, Mianyang 621010, China; 2 Groupe de Recherche en Physiologie végétale (GRPV), Earth and Life Institute–Agronomy (ELI-A), Université catholique de Louvain, 1348 Louvain-la-Neuve, Belgium; 3 Key Laboratory of Biogeography and Bioresources, Xinjiang Institute of Ecology and Geography, Chinese Academy of Science, Urumqi 830011, China; 4 University of Chinese Academy of Sciences, Beijing 100039, China

## Abstract

The effects of moderate salinity on the responses of woody plants to UV-B radiation were investigated using two *Populus* species (*Populus alba* and *Populus russkii*). Under UV-B radiation, moderate salinity reduced the oxidation pressure in both species, as indicated by lower levels of cellular H_2_O_2_ and membrane peroxidation, and weakened the inhibition of photochemical efficiency expressed by O-J-I-P changes. UV-B-induced DNA lesions in chloroplast and nucleus were alleviated by salinity, which could be explained by the higher expression levels of DNA repair system genes under UV-B&salt condition, such as the PHR, DDB2, and MutSα genes. The salt-induced increase in organic osmolytes proline and glycine betaine, afforded more efficient protection against UV-B radiation. Therefore moderate salinity induced cross-tolerance to UV-B stress in poplar plants. It is thus suggested that woody plants growing in moderate salted condition would be less affected by enhanced UV-B radiation than plants growing in the absence of salt. Our results also showed that UV-B signal genes in poplar plants *PaCOP1*, *PaSTO* and *PaSTH2* were quickly responding to UV-B radiation, but not to salt. The transcripts of *PaHY5* and its downstream pathway genes (*PaCHS1*, *PaCHS4*, *PaFLS1* and *PaFLS2*) were differently up-regulated by these treatments, but the flavonoid compounds were not involved in the cross-tolerance since their concentration increased to the same extent in both UV-B and combined stresses.

According to FAO, the total saline area accounted for 20% of total earth land^1^. In numerous semi-arid or arid regions, forest ecosystems suffer from moderate salt stress. Salinity may cause both osmotic and ionic stresses to plants. Saline soil indeed reduces the plant’s ability to take up water and causes water-deficit. Excessive amounts of putatively toxic ions enter in the plant and may be translocated by the transpiration stream, ultimately causing cell injuries and numerous metabolic disorders within the plant. However, plants have evolved many strategies to alleviate salinity stress. For example, they are able to perform osmotic adjustment through the accumulation of osmolytes, such as proline, glycine betaine, and oligosaccharides^2^ in order to maintain water absorption. At the same time, plants can synthesize protective proteins and antioxidant enzymes, such as superoxide dismutase (SOD), catalase (CAT) and ascorbate peroxidase (APX), to protect cell against the various damages induced by reactive oxygen species (ROS) which are commonly overproduced under stress conditions. Moreover, plants could minimize the deleterious effects of ionic stress by exclusion of Na^+^ from leaf tissues and by compartmentalization of Na^+^ into vacuoles^3^,^4^,^5^.

In recent decades, solar ultraviolet-B radiation (UV-B) strongly increased at the earth’s surface and now affects forest ecosystem as a result of stratospheric ozone depletion caused by atmospheric pollution^6^. The high energetic UV-B photons can produce mutagenic lesions in DNA, mainly cyclobutane pyrimidine dimmers (CPDs) and pyrimidine-pyrimidone (6, 4) photoproducts (6-4 PPs)^7^. These photoproducts compromise DNA replication, RNA transcription and protein biosynthesis^8^. The production of ROS and associated oxidative damages have also been observed in plants exposed to high UV-B doses^9^,^10^. However, plants have evolved several defensive strategies to cope with high UV-B radiation. The UV-B signal response in plants is regulated by a specific pathway that involves the UV RESISTANCE LOCUS 8 (UVR8) gene^11^,^12^,^13^. UV-B light regulates the expression of a wide range of genes involved in acclimation to and protection against UV-B radiation at a transcriptional level through UVR8^14^,^15^. Such an acclimation leads to morphological modification and improvement of protective secondary metabolites synthesis in order to attenuate the consequence of UV-B radiation in sensitive leaf tissues.

Although the responses to UV-B radiation were studied in several woody plant species, the effects of UV-B radiation on plants grown on saline soil still remain poorly understood. Previous studies reported different interaction effects between UV-B stress on the one hand and osmotic or ionic stresses on the other hand. Yang *et al.*^16^ demonstrated that UV-B radiation could mitigate the deleterious impact of heavy dessiccation in sea buckthorn to some degree. In contrast, Ren *et al.*^17^ reported that UV-B reinforce the negative impact of moderate drought stress in two *Populus* species. Kovács *et al.*^18^ reported that moderate UV-B radiation could modify the acclimation processes to drought or cadmium stress in wheat, while Rojas-Lillo *et al.*^19^ found that enhanced UV-B radiation aggravated manganese (Mn^2+^) toxicity in highbush blueberry (*Vaccinium corymbosum*) cultivars. Strickler *et al.*^20^ found that low temperature could reduce eDNA (environmental DNA) degradation rates induced by UV-B radiation in aquatic microcosms bullfrog tadpoles. Puniran-Hartley *et al.*^21^ also observed that salt pretreatment improved UV-B tolerance in wheat and barley. These studies thus confirm interaction effects between UV-B radiation and osmotic or ion stresses but data remain contradictories.

In central Asia region, saline region covers over 30% land area. Soil salinisation is mainly induced by high evaporation and low annual rainfall and constitutes an increasing problem. In the forest zone located in the river basins or oasis, woody plant species are suffering from low to moderate salt stress. *Populus alba* L. var. *pyramidalis* Bunge, a variant species of *Populus alba* L. (Sect. *Populus*), and *Populus russkii* Jabl., are widely distributed in this region^22^. *Populus alba* is much more tolerant to salt stress than *P. russkii.* In the present study, these two *Populus* species exhibiting contrasting levels of salt tolerance are used to explore the physiological and molecular responses to UV-B radiation. Our aim is to clarify the effects of moderate salt stress on plant responses to UV-B radiation and to explore the underlying mechanism for the UV-B and salt interaction effects under conditions similar to those encountered by the plants in their original environment.

## Results

### Gas exchange and Chlorophyll a fluorescence

**Figure 1:**
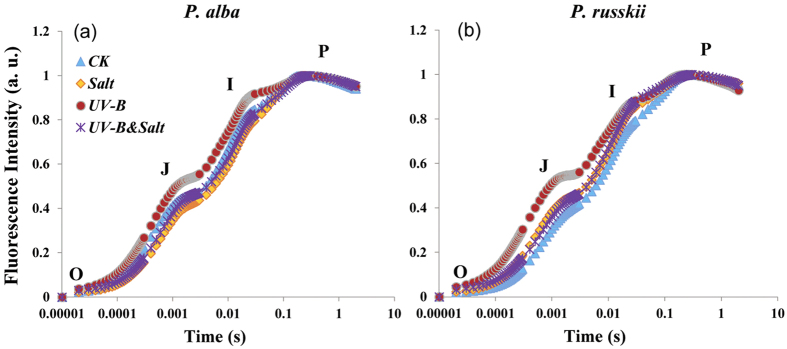
Typical OJIP chlorophyll a fluorescence transients from a healthy Control (CK, blue line), Salt treatment (orange line), UV-B radiation (red line) and UV-B&Salt treatments (purple line) plants of (**a**) *Populus alba* and (**b**) *Populus russkii*. The OJIP transients are plotted on a logarithmic time scale from 50 μs to 1 s. The mark of O, J, I and P refer to the specific time points in the JIP-test. The OJIP transients, measured in arbitrary units (a. u.), have been normalized by *F*_o_ and subjected to the measured condition as describe in “Material and Method”.

Parameters of gas exchange and chlorophyll a fluorescence are showed in Table 1 and Fig. 1. Net photosynthesis (*P*_n_) of the two species was significantly decreased by salt, UV-B and the combination of UV-B&salt (*P* < 0.05), with a high negative impact of the two last treatments. In addition, the transpiration rates (*E*) and the stomatal conductance were significantly decreased in response to salinity in the two considered species.

The changes of chlorophyll a fluorescence related parameters are listed in Table 1. The maximum quantum yield (*Φ*_Po_, *F*_v_/*F*_m_) and quantum yield of electron transport (*Φ*_Eo_) of both species were significantly decreased by UV-B or combined UV-B&salt treatment, but remained unaffected by salt treatment. In contrast, the quantum ratio for thermal dissipation of energy (*Φ*_Do_, *F*_o_/*F*_m_) increased significantly under UV-B or UV-B&salt in both species. For these chlorophyll a fluorescence parameters, UV-B&salt combination had similar effect comparatively to UV-B treatment alone. All the O-J-I-P transients were normalized at the O- and P- step for revealing more clearly changes in the shape of the transients between these two extrema. Distinct O-J-I-P changes were observed between control and UV-B in the shape of the O-J-I-P transient for both *P. alba* (Fig. 1a) and *P. russkii* (Fig. 1b). However, the differences of O-J-I-P curve between control and salt (or UV-B&salt) was observed in *P. russki* only.

### Changes in the concentration of MDA, H_2_O_2_, Proline and Glycine betaine

**Figure 2:**
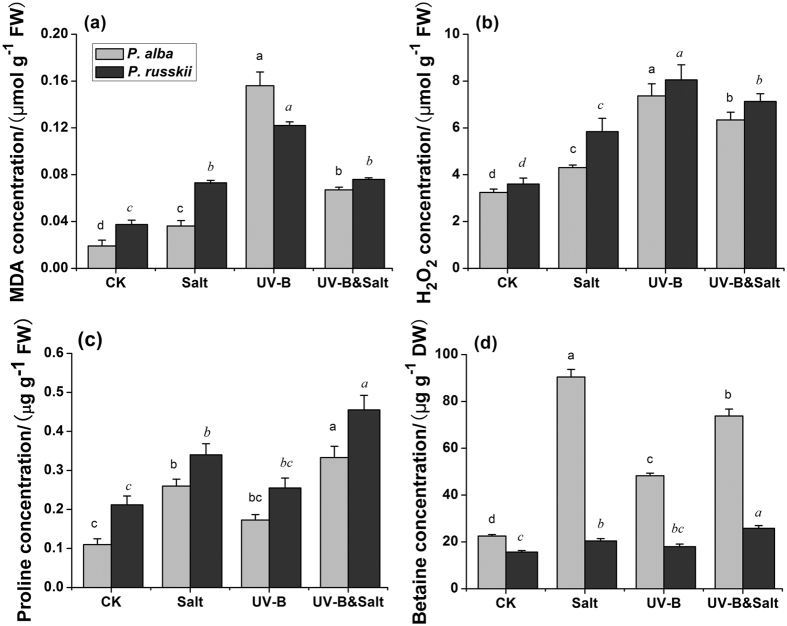
The effects of ultraviolet-B radiation (UV-B), salinity stress and their combined treatments on (**a**) malondialdehyde (MDA) concentration, (**b**) H_2_O_2_ concentration; (**c**) Proline concentration and (**d**) Glycine betaine concentration in leaves of two *Populus* species (*P. alba* and *P. russkii*). Data shown are the average mean ± *SE* of three replicates (*n* = 3). Different letters in the same font-style (non-italic and italic) indicated statistical significance at the *P* < 0.05 level among different treatments according to Tukey’s test.

Oxidative damages in leaves of the two *Populus* species were measured in terms of malondialdehyde (MDA) and hydrogen peroxide (H_2_O_2_) concentrations. Compared with controls, the concentrations of MDA and H_2_O_2_ were significantly increased under salinity stress, UV-B radiation and combined treatment in both in *P. alba* and *P. russkii* (Fig. 2). In the leaves of the two species, however MDA and H_2_O_2_ concentrations reached a maximum under UV-B radiation and was significantly reduced when combined with salt (Fig. 2a,b).

The proline concentration in the two poplar species were significantly increased by salt as well as UV-B radiation, and further increased by UV-B&salt treatment (Fig. 2c). Glycine betaine accumulated to higher extent in *P. alba* than in *P. russkii*, especially in response to salt treatment (Fig. 2d).

### Changes in antioxidant enzymes activities

**Figure 3:**
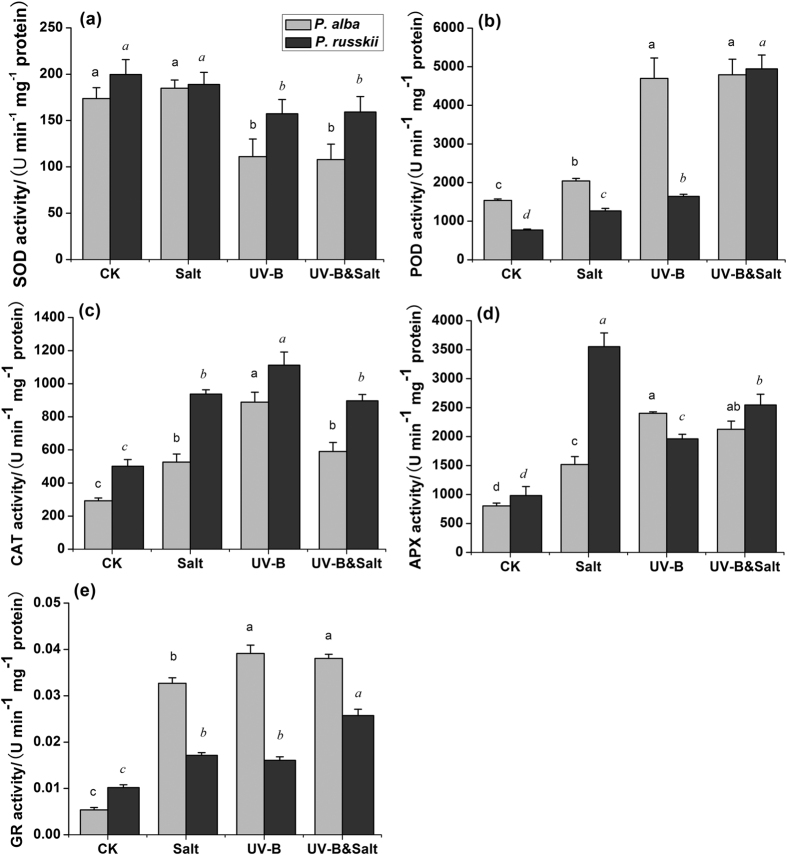
The effects of ultraviolet-B radiation (UV-B), salinity stress and their combined treatments on the antioxidant enzymes activities in leaves of two *Populus* species (*P. alba* and *P. russkii*). (**a**) Superoxide dismutase (SOD) activity; (**b**) Guaiacol peroxidase (POD) activity; (**c**) Catalase (CAT) activity; (**d**) Ascorbate peroxidase (APX) and (**e**) Glutathione reductase (GR) activities. Data shown are the average mean ± *SE* of three replicates (*n* = 3). Different letters in the same font-style (non-italic and italic) indicated statistical significance at the *P* < 0.05 level among different treatments according to Tukey’s test.

Salt, UV-B and UV-B&salt significantly enhanced antioxidant enzymes activities such as guaiacol peroxidase (POD), catalase (CAT), ascorbate peroxidase (APX) and glutathione reductase (GR) in the two *Populus* species (Fig. 3). The recorded increases in POD and GR activities were higher in *P. alba* than in *P. russkii* whatever the treatments. In comparison with UV-B stress condition (Fig. 3b,e; Table S2), the UV-B&salt combination induced higher or similar POD, APX and GR activities, but lower CAT activities. The superoxide dismutase (SOD) activity in the two poplar species remained unchanged under salinity stress, but decreased in response to UV-B and combined treatments (Fig. 3a; Table S2).

### Changes in the concentration of flavonols compounds and anthocyanins

**Figure 4:**
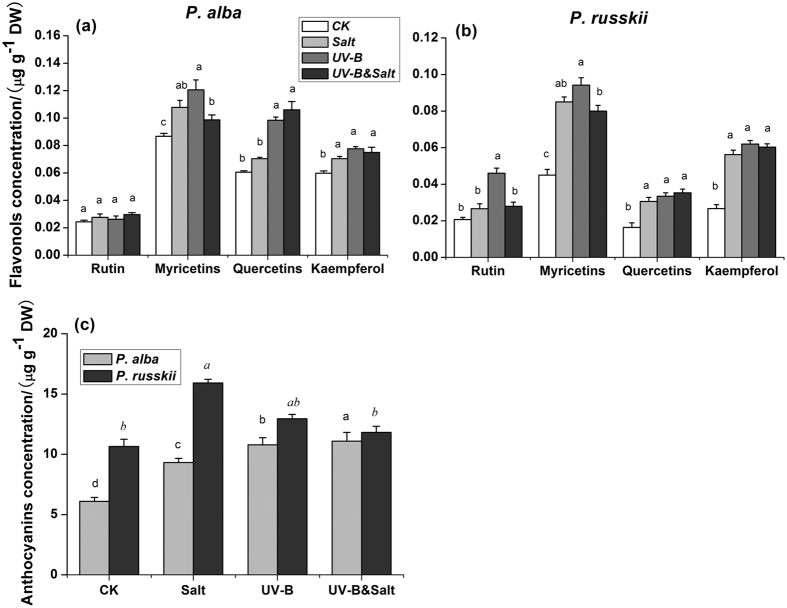
The effects of ultraviolet-B radiation (UV-B), salinity stress and their combined treatments on the concentrations of flavonols and anthocyanins in leaves of two *Populus* species (*P. alba* and *P. russkii*). (**a**) Flavonols concentration of *P. alba*; (**b**) Flavonols concentration of *P. russkii*; (**c**) Anthocyanins concentration. Data shown are the average mean ± *SE* of three replicates (*n* = 3). Different letters in the same font-style (non-italic and italic) indicated statistical significance at the *P* < 0.05 level among different treatments according to Tukey’s test.

A total of four flavonols components were detected in poplar leaves, including myricetin, quercetin, kaempferol and rutin. Generally those four flavonols components significantly increased in response to salt, UV-B and UV-B&salt combination except rutin in *P. alba* (Fig. 4a,b; Table S2). The concentrations of these compounds showed the highest value under UV-B radiation or UV-B&salt combined stress condition in both species. Although concentrations of the flavonols compounds in *P. russkii* were lower than in *P. alba* under control condition, they markedly increased to a higher extent under stress conditions. The concentration of total anthocyanins in *P. alba* was significantly increased by salt, UV-B radiation and UV-B&salt combination (Fig. 4c; Table S2), while it was increased only by salt condition in *P. russkii*.

### Dynamic transcript of genes for UV-B signaling and phenylpropanoid content synthesis

Since our results showed that the physiological and biochemical responses were almost similar in the two studied *Populus* species, we conducted the gene transcript abundance analyses using *Populus alba* only.

**Figure 5:**
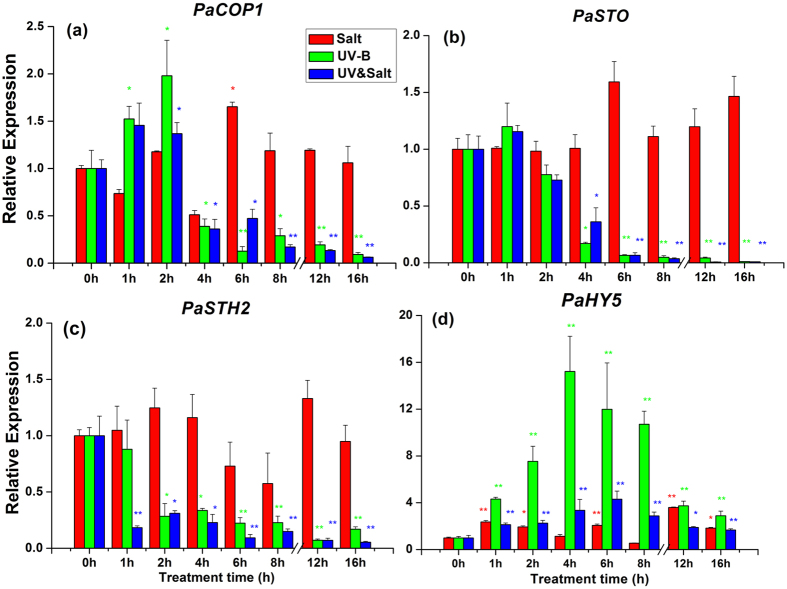
The transcript abundance of (**a**) *PaCOP1,* (**b**) *PaSTO*, (**c**) *PaSTH2* and (**d**) *PaHY5* in leaf of *Populus alba* under Salt, UV-B radiation and UV-B&Salt treatment for different treatment duration. 0 h, 1 h, 2 h, 4 h, 6 h and 8 h means the UV-B radiation and salt treatment duration was 0 h, 1 h, 2 h, 4 h, 6 h and 8 h respectively, 12 h and 16 h means 8 h treatment duration (UV-B) in the first day plus the another 4 h and 8 h treatment duration separately in the second day. The transcript abundance in the control treatment (0h) was set at 1. Data shown are the average mean ± *SE* of three replicates (*n* = 3). Different asterisks indicated statistical significance among different treatment duration according to Tukey’s test, * in present at the *P* < 0.05 level, ** in present at the *P* < 0.01 level.

It was showed in Fig. 5 for the dynamic transcript changes for gene involved in UV-B perception and signal transduction pathway, such as ELONGATED HYPOCOTYL5 (HY5), CONSTITUTIVELY PHOTOMORPHOGENIC1 (COP1), SALT TOLERANCE/B-BOX ZINC FINGER PROTEIN24 (STO/BBX24) and SALT TOLERANCE HOMOLOG2 (STH2)^23^,^24^,^25^,^26^. *PaCOP1* was up-regulated within the first two hours of UV-B treatment and then decreased. A similar trend was observed for UV-B&salt treatment. In contrast, only minor changes occurred for *PaCOP1* transcript abundance during 0–16 h salt treatment except for 4 h in (Fig. 5a). *PaSTO* and *PaSTH2* transcripts abundance pronouncedly and continuously decreased after 2–4 h of UV-B treatment or UV-B&salt treatments (Fig. 5b,c). They fluctuated to a low extent (less than 1 fold) in response to salt treatment alone (Fig. 5c). The transcription factor *PaHY5* transcript abundance presented a noticeable increase under UV-B treatment or UV-B&salt combination, and reached a peak at 4–6 h (Fig. 5d). Salt treatment alone also increased *PaHY5* transcript abundance over 1 fold except for 4 h and 8 h.

**Figure 6:**
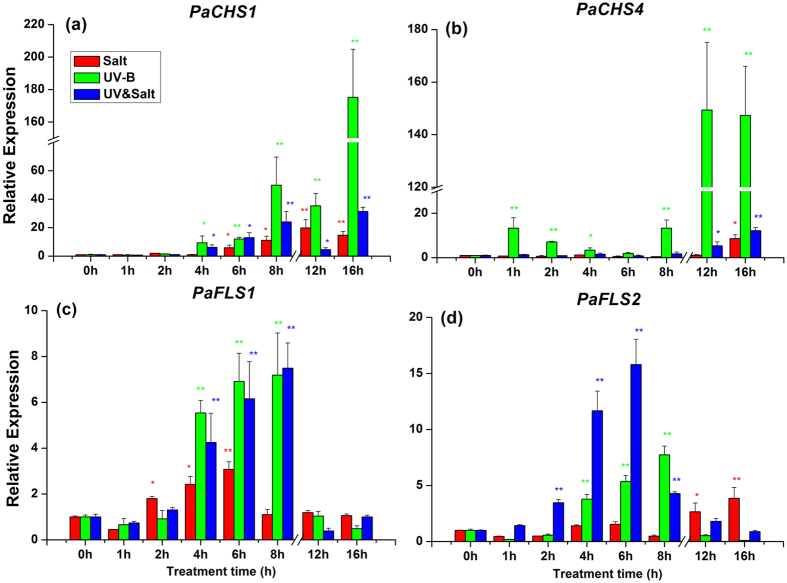
The transcript abundance of (**a**) *PaCHS1,* (**b**) *PaCHS4,* (**c**) *PaFLS1* and (**d**) *PaFLS2* in leaf of *Populus alba* under Salt, UV-B radiation and UV-B&Salt treatment for different treatment duration. 0 h, 1 h, 2 h, 4 h, 6 h and 8 h means the UV-B radiation and salt treatment duration was 0 h, 1 h, 2 h, 4 h, 6 h and 8 h respectively, 12 h and 16 h means 8 h treatment duration (UV-B) in the first day plus the another 4 h and 8 h treatment duration separately in the second day. The transcript abundance in the control treatment (0 h) was set at 1. Data shown are the average mean ± *SE* of three replicates (*n* = 3). Different asterisks indicated statistical significance among different treatment duration according to Tukey’s test, * in present at the *P* < 0.05 level, ** in present at the *P* < 0.01 level.

In our study, *PaCHS1* and *PaCHS4* (Chalcone synthase) were over-expressed in response to salt, UV-B and UV-B&salt treatments. This was especially the case for *PaCHS4* transcript abundance was increased to a higher extent in response to UV-B treatment than other treatments (Fig. 6a,b). *PaFLS1* and *PaFLS2* (Flavonol synthase) transcript exhibited a prominent increase in response to UV-B treatment or UV-B&salt combination, reaching a peak after 6–8 h and then gradually decreasing to their initial level (Fig. 6c,d). Salinity had a lower impact than other treatments on *PaFLS1* transcript abundance while *PaFLS2* was increased over 1 fold after 12–16 h of under salt treatment. The different treatments had no effect on the transcript abundance of *PaCHI* (Chalcone isomerase) (Fig. S2).

### Dynamic change for DNA-repairing genes transcript and DNA lesions

**Figure 7:**
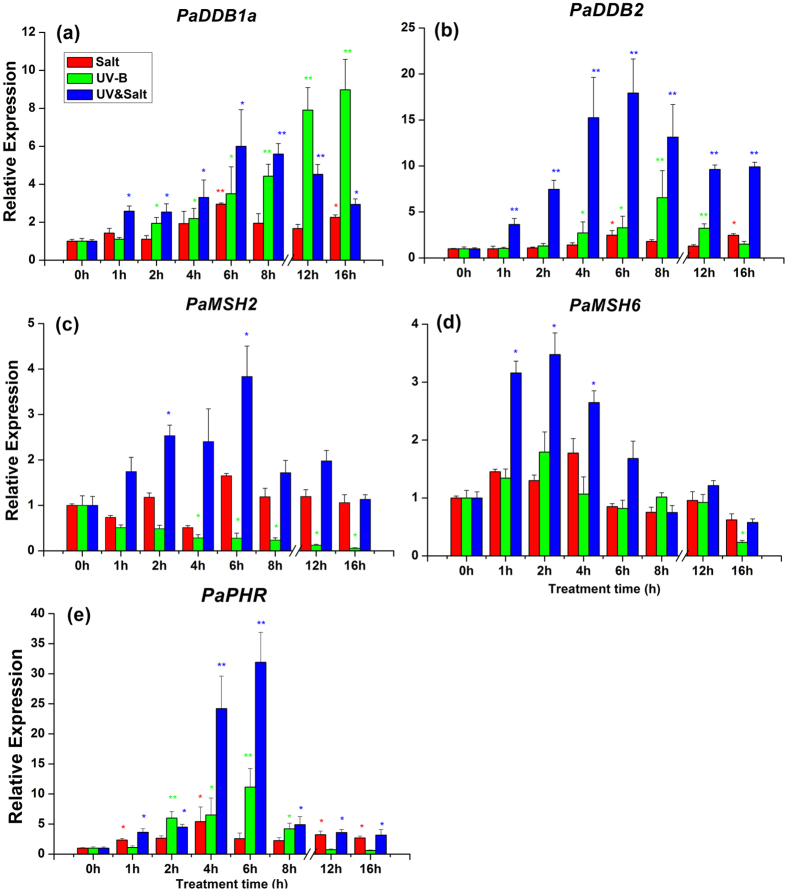
The transcript abundance of genes which were involving to damaged-DNA repairing in leaf of *Populus alba* under Salt, UV-B radiation and UV-B&Salt treatments for different treatment duration. 0 h, 1 h, 2 h, 4 h, 6 h and 8 h means the UV-B radiation and salt treatment duration was 0 h, 1 h, 2 h, 4 h, 6 h and 8 h respectively, 12 h and 16 h means 8 h treatment duration (UV-B) in the first day plus the another 4 h and 8 h treatment duration separately in the second day. (**a–e**) separately mean transcript change of *PaDDB1, PaDDB2, PaMSH2, PaMSH6* and *PaPHR* gene. The transcript abundance in the control treatment (0 h) was set at 1. Data shown are the average mean ± *SE* of three replicates (*n* = 3). Different asterisks indicated statistical significance among different treatment duration according to Tukey’s test, * in present at the *P* < 0.05 level, ** in present at the *P* < 0.01 level.

In the present study, the transcript changes for genes involved in DNA repair processes were examined focusing on genes coding for the Damaged-DNA binding protein complex (DDB) (*PaDDB1a, PaDDB1b, PaDDB2*), MMR system (*PaMSH2, PaMSH6*) and DNA photolyases (*PaPHR*). *PaDDB1a* transcript level was prominently and consistently increased by UV-B radiation, and its abundance under salt or UV-B&salt combination was also increased, reaching a peak after 6–8 h (Fig. 7a). Salinity and UV-B had no impact on *PaDDB1b* expression, whatever the duration of exposure (Fig. S3). *PaDDB2* transcription exhibited a significant increment under UV-B and UV-B&salt combination, and reached a high expression level after 6–8 h (Fig. 7b). *PaDDB2* was also significantly induced by salinity between 6 and 16 h of treatment (Fig. 7b). Although *PaMSH2* transcript abundance was gradually decreased by exposure to UV-B radiation alone (Fig. 7c), it was up-regulated by UV-B&salt treatment and peaked at 6 h. The transcripts corresponding to *PaPHR* and *PaMSH6* were up-regulated by all treatments and peaked at 2–6 h (Fig. 7d,e). The transcript abundances of *PaDDB2, PaMSH2, PaMSH6* were much higher in plants exposed to the combined UV-B&salt treatment than in those exposed to UV-B radiation alone at almost at all time points. A similar trend was also observed for *PaPHR* transcripts for long term exposure (Fig. 7e).

**Figure 8:**
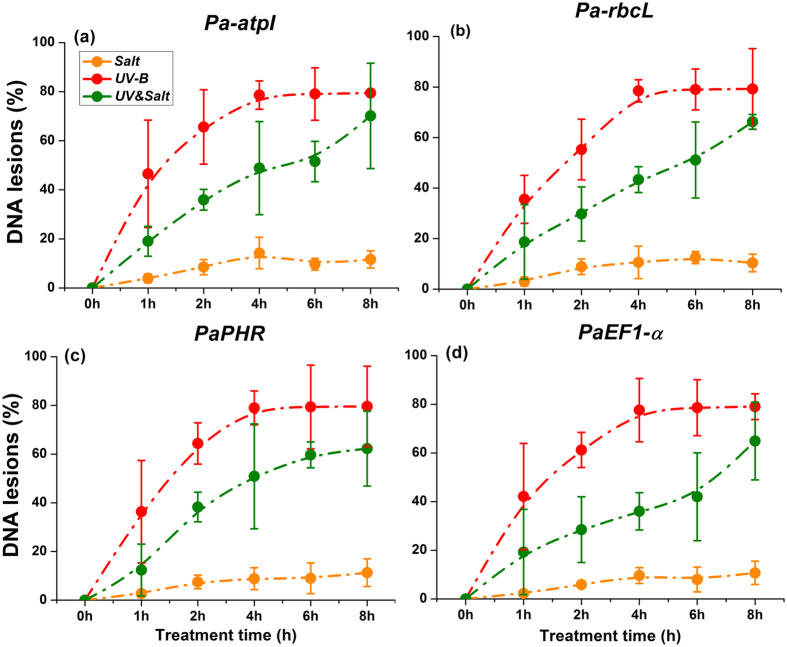
The degrees of DNA lesions in chloroplast and nuclei of *Populus alba* leaf under Salt, UV-B radiation and UV-B&Salt treatments. Chloroplast genes including (**a**) *Pa-atpI* (ATP synthase CF0 A subunit) and (**b**) *Pa-rbcL* (rubisco large subunit), nuclei genes including (**c**) *PaPHR* (CPD photolyases) and (**d**) *PaEF1-α* (elongation factor-1 alpha) in *P. alba*. 0 h, 1 h, 2 h, 4 h, 6 h and 8 h means the UV-B radiation and salt treatment duration was 0 h, 1 h, 2 h, 4 h, 6 h and 8 h respectively, 12 h and 16 h means 8 h treatment duration (UV-B) in the first day plus the another 4 h and 8 h treatment duration separately in the second day. The replication abundance in the control treatment (0 h) was set at 1. Data shown are the average mean ± *SE* of three replicates (*n* = 3).

The percentage of DNA lesions was showed in Fig. 8 for the chloroplast genes, atpI (ATP synthase CF0 A subunit) and rbcL (rubisco large subunit), as well as nuclei genes, *PaPHR* and *PaEF1**-**α* (elongation factor-1 alpha). The percentage of DNA fragments with lesions increased continuously with treatment duration for UV-B radiation as well as for the combined UV-B&salt treatment (Fig. 8). Salt treatment alone only caused a slight increase in DNA lesions percentage. However, it is noteworthy that the DNA lesions percentage in poplar plants exposed to UV-B&salt conditions was obviously lower than in the case of exposure to UV-B radiation alone for both chloroplasts and nuclear considered genes.

## Discussion

In the present work, UV-B radiation severely affected both poplar species in the absence and in the presence of NaCl as indicated by the changes of net photosynthesis and chlorophyll a fluorescence-related parameters (Table 1). *Populus russkii* was more affected by salt stress than *P. alba* in terms of photosynthetic-related parameters but we observed on little difference between the two poplar species for plant responses to UV-B radiation or combined stresses (Table 1, Table S2; Figs 1). Therefore, quantitative PCR analysis and DNA damage quantification were analyzed on one poplar species in order to decipher the complex interplay of poplar transcript profile under salt, UV-B and their combined stresses.

### Specific effects of salt stress or UV-B radiation for poplar plants

Our research confirmed that poplar plants were exposed to both ionic and osmotic stresses under moderate salinity treatment (100 mM NaCl) for 15 days (Fig. S1). Under our experimental conditions, net photosynthesis was significantly reduced in relation to *g*_s_ decrease but the different parameters of chlorophyll a fluorescence remained unchanged (Table 1, Fig. 1), suggesting that the osmotic component of stress occurred before ionic stress. In addition, salt-stressed plants suffered only from a moderate oxidative pressure, as indicated by the moderate increase of cellular H_2_O_2_ and MDA contents and little change of SOD activity (Figs 2 and 3). The O-J-I-P transient kinetics has been employed to represent the successive reduction of electron transport pool of PSII^27^, and according to this criteria salt stress symptom was only observed in the salt sensitive poplar species *P. russkii* (Fig. 1).

Organic osmolytes content such as proline and glycine betaine are known to be directly involved in protection of cellular structures and regulation of the osmotic balance^28^. The rapid increase of glycine betaine concentration in the salt-tolerant poplar species *Populus alba* could explain its higher resistance to salt stress comparatively to *P. russkii* (Fig. 2). In addition, UV-B induced increase of glycine betaine synthesis was detected in *P. alba*, but to the best of our knowledge has not been reported yet in other plant species, which could further explain that *P. alba* is able to cope with environmental stresses through the synthesis of protective organic osmolytes.

On the other hand, the poplar plants were more severely affected by UV-B radiation than by moderate salt stress. This could, at least partly be linked to a reduced sunshine intensity in greenhouse, which rendered the plants more susceptible to subsequent burst of UV-B radiation. According to our data, osmotic stress occurred in UV-B stressed plants, as suggested by increase in concentrations of organic osmolytes such as proline and glycine betaine, which were consistent with previous studies^29^,^30^. In spite of UV-B-caused osmotic stress, the deleterious effect of UV-B on poplar plants was primarily ascribed to the oxidative damage induced by excessive ROS production, as indicated by prominent enhancement of cellular H_2_O_2_ and MDA concentration (Fig. 2). The reduced SOD activity under UV-B irradiation further aggravated ROS pressure under UV-B stress, which showed that ROS balance was harmed in some extent. Previous studies showed that SOD activity was inhibited under high UV-B intensity or other stress^31^,^32^,^33^,^34^.

The reduced chlorophyll fluorescence-related parameters and modification in the O-J-I-P curve showed that UV-B stressed poplar plants were affected in their photochemical properties. The O-J-I-P fluorescence transients revealed that the damage caused by UV-B irradiation was prominent at the donor side of PSII. This result was consistent with previous studies in other plants^23^,^31^. The oxidative pressures/stress evoked by UV-B irradiation or salt stress has provoked the increase in antioxidative enzymes activities in order to scavenge ROS overproduced under UV-B exposure or salt stress^35^,^36^,^37^, such as POD, CAT, APX and GR etc. (Fig. 3).

UV-B light serves as an important factor to regulate genes expression involved in the light signaling pathway^25^,^38^. Unfortunately, data concerning woody plants remain scarce. As a E3 ubiquitin ligase, CONSTITUTIVE PHOTOMORPHOGENESIS1 (COP1) represses photomorphogenesis by promoting degradation of the transcription factors, ELONGATED HYPOCOTYL5 (HY5). In the presence of UV-B radiation, COP1 interact with UVR8 monomer leading to HY5 stabilisation and allowing activation of UV-B-responsive genes. Huang *et al.*^39^ reported that COP1 is UV-B inducible, and that HY5 promotes COP1 expression via a positive feedback loop. Our results showed that *PaCOP1* and *PaHY5* were up-regulated in poplar when exposed to UV-B radiation. After enhancement of HY5 transcript, COP1 transcript was significantly reduced at the end of stress exposure. We hypothesize that this process could avoid HY5 degradation when HY5 protein was abundant. The dynamic transcript change of *PaCOP1* of *PaHY5* showed that poplar plants quickly responded to UV-B signal. Huang *et al.*^39^ also observed down-regulation of COP1 transcript in *Arabidopsis* after 4 hours of UV-B onsets. SALT TOLERANCE (STO/BBX24) was defined as a new signaling component that has a negative role in the UV-B-mediated photomorphogenesis by interacting with COP1 and repressing HY5 transcriptional activity in *Arabidopsis*^26^. Down-regulation of *PaSTO* transcript and its homologous genes, *PaSTH2*, play a protective role against UV-B radiation in poplar plants. Lippuner *et al.*^40^ demonstrated that the STO gene expression could not be induced by salt treatment, which is in agreement with our own results.

Key genes of phenylpropanoid biosynthesis pathway act downstream of *PaHY5*, and include *PaCHS1*, *PaCHS4*, *PaFLS1* and *PaFLS2.* Their transcripts sensitively responded to UV-B, salt and other ROS stress (Fig. 6). Previous studies reported that the expression level of CHS genes exhibit a positive correlation with UV-B intensity or ROS synthesis^41^,^42^. The dynamic transcript change of these genes in leaves of *P. alba* may thus explain the increased concentration of flavonols (such as myricetin, quercetin, kaempferol and rutin) or anthocyanins, which play a crucially role in protecting plants against UV-B damages or ROS stress^43^,^44^,^45^.

DNA lesions could be caused directly by UV-B radiation or indirectly generated by unquenched ROS under environmental stresses^46^. DNA lesions were estimated by quantitative PCR (qPCR) with genome-specific primers to measure polymerase-blocking lesions^8^. The qPCR was blocked when DNA polymerase encounters photoproducts or other lesions in the DNA template. The percentage of DNA fragments with one or more lesions was measured by the frequency that encountered DNA lesions in replication. To the best of our knowledge, this is the first time that this method is used in a woody plant species. Our results showed that this method is highly sensitive for detection of DNA lesions in nucleus or chloroplast (Fig. 8). Indeed, even the slight DNA lesions caused by a moderate salt treatment could be detected.

The DNA lesion rate was observed in the presence of UV-B radiation and UV-B&salt interaction. To repair DNA lesions maintain DNA integrity, it is crucial to activate DNA repair system, including the photorepair pathway mediated by photolyase and the dark repair pathway mediated by nucleotide excision repair (NER)^8^,^47^. Cyclobutane pyrimidine dimer (CPD) photolyase is an essential repair enzyme for UV-B-induced CPDs^48^. An increase *PaPHR* transcript implies that this gene is also functioning in excising DNA lesion caused by ROS in poplar plants (Fig. 7). Damaged-DNA binding protein complex (DDB) could distinguish DNA lesions^49^,^50^, and DDB2 binds to DDB1 to form the UV-damaged DNA-binding protein complex and recruit proteins of the nucleotide excision repair pathway (the NER pathway) to trigger DNA repair. In our study, the recorded increase in DDB1a and DDB2 transcripts showed that poplar plants responded sensitively to UV-B or salt stress. Previous studies have confirmed that the DDB1 and DDB2 gene were regulated by ROS^51^,^52^, and this is confirmed by our results (Fig. 7). MSH2 and MSH6 are important components of the post-replicative DNA mismatch repair system (MMR). They form a heterodimer that binds to DNA mismatches thereby initiating DNA repair process. In our study moderate up-regulation of MSH6 under UV-B or salt stress was accompanied by down-regulation of MSH2 by UV-B, showing that modalities MSH2/MSH6 complex formation may be differentially regulated by distinct environment cues. Previous study also showed that the transcripts of MSH2 and MSH6 were variously regulated by UV-B stress, but the underlying mechanism is not clear yet.

### Modification of moderate salt stress on UV-B responses in poplar plants

In our study, a moderate salinity treatment clearly mitigated the detrimental effects caused by UV-B radiation on poplar plant species. Inhibited photochemical function by UV-B radiation was alleviated by salt treatment, since O-J-I-P test showed that high J-I peak value caused by UV-B radiation was reduced when salt treatment was added (Fig. 1). Meanwhile, UV-B-induced high cellular ROS pressure, as showed by H_2_O_2_ content and MDA contents, was also reduced by the imposed salt treatment (Fig. 2). A similar trend was also observed for DNA lesions (Fig. 8). The beneficial effects of moderate salt treatment on the UV-B stressed plants could be referred as cross-tolerance process which implies that an organism exposed to one stress is able to increase its tolerance to another one^53^,^54^.

The cross-tolerance effects could be due to the synergistic co-activation of non-specific stress-responsive pathways sharing common components and triggering physiological processes involved in the plant response to different environmental constraints^55^. It was demonstrated salt treatment exerted major protective effects under combined stressed by significant UV-B&salt interaction effects of glycine betaine, CAT activity and flavonol compounds (Table S2), which led to lower MDA and H_2_O_2_ concentrations in combined stresses than UV-B alone and performed as cross-tolerance. In our study, more efficient synthesis of protecting compatible organic solutes by salt treatment, such as proline and glycine betaine compound, may contribute to tolerance to UV-B radiation. Beside its well-known function in maintaining cellular osmosis potential under salt or drought stress, proline is also known as an effective involved in ROS quenching. In addition, Shabala *et al.*^56^ reported that exogenous application of glycine betaine alleviated UV-B-caused damage to PSII in quinoa leaf mesophyll, and is highly efficient to prevent hydroxyl radical-induced disturbance of intracellular potassium homeostasis in roots^57^. Recently Puniran-Hartley *et al.*^21^ reported that salinity-induced accumulation of organic osmolytes in barley and wheat leaves improved PS II activity and oxidative stress tolerance in the presence of UV-B radiation. Furthermore, significant UV-B&salt interaction effects in APX and GR activity and anthocyanins concentration showed that UV-B or salt offered cross-tolerance against the other stress under combined stresses.

Secondly, transcript of genes for the DNA repair process such as *PaDDB2*, *PaMSH6* and *PaPHR* increased to a higher extent in response to combined stress than in response to UV-B alone (Fig. 7). This may afford a more efficient protection against DNA damage than UV-B alone. Moreover, down-regulation of *PaMSH2* transcript under UV-B radiation was reversed by salt treatment and those transcripts increased in response to combined stress, which could afford more effective DNA mismatch repair (MMR) ability. As a consequence, DNA from plants exposed to combined stress suffered lower ROS pressure than those exposed to UV-B alone (Fig. 2). A similar observation is also valid for reduced DNA lesion. The internal mechanism for the alleviation of DNA damage merits further exploration.

Gene expression of UV-B signaling pathway (*PaHY5*, *PaCOP1*, *PaSTO* and *PaSTH2*) exhibited similar pattern under UV-B stress alone and under the combined treatment. In contrast, *PaCHS1* expression pattern in plants exposed to UV-B&salt appeared similar with those recorded in salt-treated plants (Fig. 6a). Moreover, the transcript pattern of *PaFLS1* and *PaFLS2* gene in UV-B&salt interaction appeared to be mediated by both UV-B and salt treatment. The CHS enzyme is a rate-limiting enzyme for the synthesis of precursor of flavonols and anthocyanins^58^, while genes from FLS family are encoding a key component controlling the last step of flavonol synthesis^38^,^59^. The change pattern of CHS and FLS genes transcripts led to the different concentrations of various flavonols components and anthocyanins (Fig. 4a–c), but only minor differences were recorded between UV-B treatment and its combination with salt. Therefore the phenylpropanoid pathway had little relation with the alleviation of UV-B damage by salt, at least in terms of gene activation.

In conclusion, moderate salt treatment alone (100 mM NaCl) affected *P. russkii* more than *P. alba*. Plant tolerance to UV-B radiation was improved by moderate salinity in the two studied poplar species, and only little inter-specific differences were observed when plants were exposed to UV-B and salt combination. Poplar trees exposed to combined stresses exhibited lower levels of cellular H_2_O_2_ and membrane peroxidation than trees exposed to UV-B alone. The deleterious impact of UV-B radiation on photochemical efficiency was also reduced under salinity conditions. UV-B-induced DNA lesions in chloroplast and nucleus were alleviated by 100 mM NaCl, which could be related with a more efficient system for DNA repair. The transcripts of genes involved in DNA repair process such as *PaPHR*, *PaDDB2*, *PaMSH2* and *PaMSH6* were significantly enhanced by salt treatment. Similarly, higher concentrations of protecting organic osmolyte, e.g. proline and glycine betaine, afforded more effective protection under combined stress than in response to UV-B alone.

As a consequence, salt treatment appeared to induce a cross-tolerance to UV-B stress, and the woody plant species growing in moderate salinity region could be, from a relative point of view, less affected by enhanced UV-B radiation linked to depletion of stratospheric ozone than those growing in non-salted areas. On the other hand, our results showed that UV-B signal genes, such as *COP1, STO* and *STH2*, were efficiently regulated by UV-B radiation in poplar trees, but were not regulated by salt stress. Salinity, UV-B or their combination increased the transcripts corresponding to the *PaHY5* transcription factor and its downstream genes for phenylpropanoid compound synthesis, such as *PaCHS1*, *PaCHS4*, *PaFLS1* and *PaFLS2*, which led to enhanced levels of flavonol and anthocyanin compounds in stressed plants.

## Methods

### Plant material and experimental design

Cuttings of *P. alba* and *P. russkii* were collected in their natural habitat (44.29°N, 87.93°E, 200 m altitude) in North Xinjiang, Northwest China, with 80–120 mmol of soil salinity in the sampled area. The local ambient daily UV-B dose in summer was approximately 8.5 kJ m^−2^ day^−1^ according to a mathematical model by Madronich *et al.*^60^. The mean annual rainfall, annual evaporation, annual average temperature, annual maximum temperature and annual minimum temperature in this region are 164 mm, 2000 mm, 6.6 °C, 42.6 °C and −41.6 °C respectively. In spring, healthy cuttings with a uniform size were collected and transplanted into 10 L plastic pots filled with homogenized soil (1:1 sand:yellow soil v/v) and grown in a naturally lit glass greenhouse. Potted cuttings were well irrigated according to evaporation demand and watered with 1 L half-strength Hoagland nutrient solution every week. After two weeks, all of those cuttings were rooting and sprouting. The glass of greenhouse absorbed all of ambient solar UV-B radiation. Photosynthetic photon flux density (PPFD) density measured around solar noon at the top of the plants ranged from 350 μmol m^−2^ s^−1^ on cloudy days to 1760 μmol m^−2^ s^−2^ on sunny days. Particularly, sunshade net was used when the greenhouse temperature exceeded 35 °C, and this could make the PPFD decreased to one fourth, about 180–400 μmol m^−2^ s^−1^, compared with full sunshine condition.

Treatments were applied 50 days after transplanting, with a mean height of 38 cm for *P. alba.* and 50 cm for *P. russikii*. The experimental scheme had a factorial design to study the combination of two factors: UV-B radiation and soil salinity, in the two species of *Populus*. Therefore, there were four treatments for each *Populus* species: (1) no UV-B radiation and no-salinity (CK), (2) UV-B radiation and no-salinity (UV-B), (3) no UV-B radiation and salt stress (Salt), (4) UV-B radiation and salt stress (UV-B&salt). Each treatment in a *Populus* species consisted in three biological replications with four plants per replicate. The saline soils were watered with half-strength Hoagland solution containing 100 mM NaCl (watered every five days with 1 L, simulated with the soil salinity in their natural habitat), and non-saline soils were watered with half-strength Hoagland solution without any additional salt (NaCl). For UV-B treatments, plants were exposed to UV-B radiation for 8 h/day (from 09:00 to 17:00) from UV fluorescent lamps (ranging from 278 to 380 nm with a peak at 308 nm, Beijing Electronic Resource, Inc., Beijing, China) in greenhouse and placed 80 cm above plants. The total UV-B radiation intensity was measured with a UV spectra radiometer (Beijing Normal University, Beijing, China), and the UV radiometer was calibrated by USB2000 Fiber Optic spectrometer (Ocean Optics Inc., USA). The biologically effective UV-B intensity (UV-B_BE_) was calculated according to the generalized plant response action spectrum^61^ and the UV spectra (Fig. S4), the UV-B_BE_ intensity was normalized at 300 nm to obtain 0.2 W m^−2^ s^−1^ of UV-B_BE_ intensity, with 8.5 kJ m^–2^ day^–1^ of an average daily biologically effective UV-B radiation, simulating the summer solar UV-B in the natural habitat for those sampled *Populus* species. Plants that did not receive UV-B treatment were exposed to the light source described above and the lamps were covered with 0.13 mm polyester filters which absorb all radiation below 320 nm to exclude UV-B radiation.

After 15 days of treatment with UV-B radiation and salinity stress, the photosynthetic gas exchange and chlorophyll fluorescence characteristics were measured for the fourth fully expanded leaf of each plant from the apex. At the end of the experiment (15 days), the fourth to sixth fully expanded leaves were collected and frozen immediately in liquid nitrogen and stored at −80 °C prior to further processing for the measurements of physiological and biochemical parameters including antioxidant enzymes (such as SOD, POD, CAT, APX and GR), osmoprotectants (such as proline, glycine betain) and MDA, as well as determination of flavonols and anthocyanins concentrations.

To investigate molecular response of *Populus* exposed to UV-B radiation and/or salt stress, we conducted the gene transcript abundance analyses using *P. alba* only. The experiment was separately conducted immediately after the above experiment. The experimental design and the treatment methods were the same as above, the same size of *P. alba* cuttings (with no stress treatment) were applied for salinity and/or UV-B treatment, but UV-B radiation intensity was increased to 1.3 W m^−2^ for 8 h/day. Six sampling time points were considered: in the first day, sampling after 1 h, 2 h, 4 h, 6 h and 8 h of exposure to UV-B radiation/salinity stress; in the second day, sampling after 4 h UV-B radiation (totally 12 h UV-B/28 h for salinity stress), 8 h UV-B radiation (totally 16 h UV-B/32 h for salinity stress). The fourth to sixth fully expanded leaves were collected, frozen immediately in liquid nitrogen, and stored at −80 °C for gene transcript abundance analyses and DNA lesions analyses.

### Gas exchange and Chlorophyll Fluorescence measurements

Net photosynthetic rate (*P*_n_), stomatal conductance (*g*_s_), transpiration rate (*E*) and intercellular CO_2_ concentration (*C*_i_) were measured with a portable photosynthesis system (LI 6400, Li-Cor, Lincoln, NE, USA). The measurements were performed on cloudy-free days between 09:00 and 11:00 h at local time. The fourth fully expanded leaf of each plant was measured under following conditions: leaf temperature, 25 °C; saturated photosynthetic photon flux density (PPFD), 1000 μmol m^–2^ s^–1^; and ambient CO_2_ concentration, 350 ± 5 μmol mol^–1^. Chlorophyll a (Chl a) fluorescence was measured between 9:00 and 11:00 a.m. using portable plant efficiency analyzer (M-PEA, Hansatech Instruments Ltd., UK) on the same leaves where *P*_n_ was measured. Leaf clips for dark adaptation were placed on the adaxial side of the leaves for 20 min before measurement at saturating flash of 3000 μmol m^−2^ s^−1^. The Chl a fluorescence emission induced by the strong light pulses was measured and digitised between 10 μs and 1 s by the instrument. The original data such as *F*_*o*_ (fluorescence intensity at 20 μs), *F*_*m*_ (the maximum fluorescence intensity), *K*-step and *J*-step (fluorescence intensities at 300 μs and 2 ms, respectively) were measured for different treatments. Chlorophyll a fluorescence transients were quantified according to the JIP test^62^ by utilizing the original data.

### Determination of Proline, Glycine betaine, Hydrogen peroxide (H_2_O_2_), Malondialdehyde (MDA) and Na^+^ concentration

Proline was assayed as described by Bates *et al.*^63^. The exposed, fully expanded leaves (0.5 g, fresh weight) were homogenized in 5 mL of 3% sulphosalicyclic acid solution. After centrifugation, 2 mL of supernatant, 2 mL of glacial actetic acid and 2 mL of 2.5% acid ninhydrin solution were added to a test tube and covered with Teflon cap. Absorbance of the free proline concentration was measured at 520 nm and was expressed as μg g^−1^ FW. The glycine betaine content was measured using the *Plant Betaine content kit* (*Cominbio, Suzhou, China*) according to the manufacturer’s protocol. Absorbance of the glycine betaine concentration was measured at 525 nm and expressed as μg g^−1^ dry plant weight (DW). The concentration of H_2_O_2_ in poplar leaves was calculated from the absorbance of the titanium-hydroperoxide complex^64^. The concentration of H_2_O_2_ was determined using a standard curve plotted with known concentrations of H_2_O_2_ and expressed as μmol g^−1^ FW. The cellular membrane lipid peroxidation analysis was based on MDA concentration according to the method of Kramer *et al.*^65^. The absorbance of samples was measured at 450, 532 and 600 nm using spectrophotometer. The MDA concentration was calculated using the formula: MDA (μmol L^−1^) = 6.45 (A_532_ −A_600_) −0.56 A_450_. The Na^+^ concentration in leaves of two *Populus* was determined by an atomic absorption spectrophotometer using the method of Wang *et al.*^66^.

### Determination of antioxidant enzymes activities

For analysis of antioxidant enzymes activities, 0.5 g fresh leaves were ground in liquid nitrogen and extracted with 50 mM potassium phosphate buffer (pH 7.8) containing 0.1 mM EDTA, 1% (w/v) polyvinyl pyrrolidone (PVP), 0.1 mM phenylmethane sulfonyl fluoride (PMSF) solution and 0.2% (v/v) Triton X-100. Protein concentrations were determined according to Bradford^67^ method with bovine serum albumin (BSA) as a standard. The superoxide dismutase (SOD, EC 1.15.1.1) activity was assayed by monitoring the inhibition of photochemical reduction of nitro blue tetrazolium (NBT) as described by Giannopolitis and Ries^68^. One unit of SOD activity (EU g^−1^) was defined as the amount of enzyme required to cause 50% inhibition of the reduction of NBT as monitored at 560 nm. The guaiacol peroxidase (G-POD; EC 1.11.1.7.) activity was measured at 470 nm as described by Adam *et al.*^69^. The catalase (CAT; EC 1.11.1.6.) activity was assayed as described by Kovács *et al.*^18^. The ascorbate peroxidase (APX; EC 1.11.1.11.) activity was determined as described by Nakano and Asada^70^. The glutathione reductase (GR; EC 1.6.4.2.) activity was measured using the *Glutathione reductase activity kit* (*Cominbio, Suzhou, China*) according to the manufacturer’s protocol. The decrease in absorbance at 340 nm was monitored by spectrophotometer.

### Extraction and determination of anthocyanins and flavonols concentrations

Anthocyanin concentration of poplar leaves was determined using the method described by Downey and Rochfort^71^. Briefly, 5 mL of methanol: 1% HCl was added to 0.5 g of ground fresh tissue, followed by shaking overnight at 120 rpm. After centrifugation at 10,000 g for 10 min at 4 °C, 1 mL of distilled water was added to 1 mL of extract liquid, followed by addition of 1 mL of chloroform to remove chlorophyll. Absorption of the clear supernatant was then measured at 530 nm and the anthocyanin concentration was calculated using the molar absorbance of cyanidin-3-O-glucoside. The concentration of rutin, quercetin, myiricetin and kaempferol was determined using the method of Downey and Rochfort^71^ with some minor modifications. 500 mg of ground plant tissue was extracted with 9 mL 80% methanol, sonicated for 1 h, and allowed to stand overnight at 4 °C. The extract was centrifuged to remove debris and the supernatant dried under nitrogen. Dried samples were incubated with 3 mL of 1 N HCl at 90 °C for 2 h and extracted twice with 3 mL of ethyl acetate. Ethyl acetate extracts were pooled, dried under nitrogen, and resuspended in 5 mL of methanol, 20 μL of which were used for reverse-phase HPLC analysis as described below.

HPLC separation of flavonols from poplar leaves used Solvent A (0.1% phosphoric acid in water) and Solvent B (100% methanol) with the following gradient: 0 min, 10% B; 25 min, 50% B; 30 min, 80% B; 35 min, 80% B; 36 min, 10% B; 45 min, 18% B. The gradient was run at a flow rate of 1.0 mL min^−1^. The column temperature was maintained at 40 °C for the duration of the analysis. Samples were run on a Agilent 1260 (Agilent, CA, USA) HPLC system and were detected with a diode array detector set at 352 nm. Rutin, quercetin, myiricetin and kaempferol were used as the calibration standards for flavonol compounds quantification of samples respectively.

### RNA extraction and Gene expression analysis by qRT-PCR

Total RNA was extracted using *RNeasy Plant mini kit* (*Qiagen, Hilden, Germany*) according to the manufacturer’s instruction. Residual genomic DNA was digested with DNase I. The purity and concentration of total RNA was determined by A260/A280. RNA quality and integrity was assessed by 1.2% (w/v) agarose gel analysis. After that, quantified RNA was reverse transcribed into cDNA using the Reversal Transcription Reagent Kit (TaKaRa, Dalian, China) and oligo (dT) 18 primer. Synthesized cDNA were diluted to a final volume of 100 μL, and 1 μL was used as a template for qRT-PCR reactions.

The qRT-PCR analyses were performed in triplicate using the CFX96 real-time system (Bio-Rad, CA, USA). Sequences of the gene-specific primers are presented in Table S1. Primers for each of the genes under study were designed using the PRIMER5 software^72^ in order to amplify unique 150–200 bp products (Table S1). The *Populus* elongation factor-1 alpha (EF-1α) was selected as the appropriate reference gene to normalize gene expressions^73^. The qRT-PCR amplification conditions were carried out under the following conditions: 3 min denaturation at 95 °C; 45 cycles at 95 °C for 10 s and 58 °C for 15 s; and a melting curve protocol (plates read when increased 0.5 °C every 5 s from 65 °C to 95 °C). The levels of gene expression were evaluated by calculating the threshold cycle (Ct) based on 2^−ΔΔCt^ method and expressed by the relative quantitative variation^74^.

### Assessment of UV-induced DNA lesions by quantitative PCR

The assessment of DNA lesions was performed according to the method of Takahashi *et al.*^8^. Quantitative PCR (qPCR) analysis of UV-B-induced DNA lesions was based on the blockage of PCR polymerases by UV-induced photoproducts^8^,^75^. Genomic DNA was extracted from three biological replicates of poplar leaves using the method of Cetyltrimethylammonium bromide (CTAB). DNA samples were treated using RNase to remove traces of RNA, the concentration was determined using a Quawell Q6000 Analyzer (Quawell Technology Inc., USA), and all DNA samples’ were diluted to a final concentration of 20 ng μL^−1^ for qPCR analysis. Specific fragments of the genes in chloroplast and nucleus were amplified from *Populus* genomic DNA as described above: the size of each PCR product is 188 bp for atpI (chloroplast gene), 199 bp for rbcL (chloroplast gene), 169 bp for PHR (CPD photolyase), and 182 bp for EF1-α. Because targeted sequences that contained UV-induced polymerase-blocking lesions could not be amplified, the quantity of undamaged PCR-template DNA was calculated. The percentage of fragments with one or more UV-induced polymerase-blocking lesions was calculated as (A_0_ – A_D_)/A_0_·100 (%)^76^, where A_0_ was the quantity of PCR-template DNA extracted from non-irradiated leaves and A_D_ was the quantity of PCR-template DNA extracted from irradiated leaves.

### Statistical analyses

Statistical analyses for physiological parameters were performed using the SPSS software (version 16.0; SPSS Inc., Chicago, USA). Three-way ANOVAs were applied to analyze the effects of the *Populus* species, UV-B radiation, salinity and their interactions on physiological variables, and Tukey’s test was employed to detect possible differences between the treatments in each species. All data were checked for normality and the homogeneity of variances before ANOVAs, and log transformed to correct deviations from these assumptions when necessary.

## Additional Information

**How to cite this article**: Ma, X. *et al.* Moderate salt treatment alleviates ultraviolet-B radiation caused impairment in poplar plants. *Sci. Rep.* **6**, 32890; doi: 10.1038/srep32890 (2016).

## Supplementary Material

Supplementary Information

## Figures and Tables

**Table 1:** The effects of ultraviolet-B radiation (UV-B) and salinity stress and their combined treatments on photosynthetic (*P*_n_, *g*_s_, *E*, and *C*_i_) and Chlorophyll a fluorescence parameters (*Φ*_Po_ (*F*_v_/*F*_m_), *Φ*_Do_ (*F*_o_/*F*_m_) and *Φ*_Eo_) of two poplar species (*Populus alba* and *Populus russkii*).

Plant species	Treatment	*P*_n_ (μmol m^−2^ s^−1^)	*g*_s_ (mol m^−2^ s^−1^)	*E* (mmol m^−2^ s^−1^)	*C*_i_ (μmol mol^−1^)	*Φ*_Po_ (*F*_v_/*F*_m_)	*Φ*_Do_ (*F*_o_/*F*_m_)	*Φ*_Eo_
*P. alba*	Control	12.57 ± 0.42 a	1.008 ± 0.052 a	2.87 ± 0.69 a	355.5 ± 16.6 a	0.8494 ± 0.0015 a	0.1508 ± 0.0015 b	0.4472 ± 0.0106 ab
	Salt (NaCl 100 mM)	9.52 ± 1.02 b	0.446 ± 0.084 b	1.53 ± 0.22 b	361.0 ± 3.82 a	0.8306 ± 0.0022 a	0.1695 ± 0.0023 b	0.4776 ± 0.0062 a
	UV-B	4.95 ± 1.09 c	0.462 ± 0.037 b	1.08 ± 0.05 b	259.3 ± 5.83 b	0.7636 ± 0.0082 b	0.2365 ± 0.0082 a	0.3966 ± 0.0164 c
	UV-B&Salt	5.06 ± 0.31 c	0.443 ± 0.091 b	2.14 ± 0.06 ab	255.8 ± 0.03 b	0.767 ± 0.0162 b	0.2331 ± 0.0163 a	0.4153 ± 0.0214 bc
*P. russkii*	Control	8.41 ± 0.80 *a*	0.379 ± 0.101 *a*	1.38 ± 0.21 *a*	367.02 ± 8.19 *a*	0.8418 ± 0.0024 *a*	0.1583 ± 0.0023 *b*	0.482 ± 0.0224 *a*
	Salt (NaCl 100 mM)	5.52 ± 1.06 *b*	0.074 ± 0.022 *b*	0.41 ± 0.11 *b*	274.4 ± 30.9 *b*	0.844 ± 0.0022 *a*	0.1559 ± 0.0023 *b*	0.4959 ± 0.0078 *a*
	UV-B	4.79 ± 0.23 *b*	0.141 ± 0.024 *b*	1.08 ± 0.19 *ab*	353.0 ± 7.30 *a*	0.7526 ± 0.0137 *b*	0.2474 ± 0.0137 *a*	0.3815 ± 0.0178 *b*
	UV-B&Salt	4.77 ± 0.40 *b*	0.144 ± 0.024 *b*	1.14 ± 0.16 *a*	347.1 ± 12.3 *a*	0.7676 ± 0.0051 *b*	0.2323 ± 0.0050 *a*	0.4183 ± 0.0137 *b*
Results of three-way ANOVA								
Species (S)		17.001^***^	88.718^***^	20.462^***^	8.186^**^	0.038^NS^	0.027^NS^	0.867^NS^
UV-B (U)		62.048^***^	17.315^***^	0.859^NS^	13.785^**^	175.95^***^	175.56^***^	43.49^***^
Salt (N)		7.854^**^	26.273^***^	2.185^NS^	6.299^*^	0.006^NS^	0.09^NS^	5.11^*^
S×U		13.583^***^	4.911^*^	4.015^NS^	45.782^***^	0.468^NS^	0.462^NS^	2.19^NS^
S×N		0.01^NS^	2.645^NS^	0.608^NS^	6.835^*^	1.895^NS^	1.886^NS^	0.001^NS^
N×U		8.344^**^	24.455^***^	18.438^***^	4.088^NS^	2.185^NS^	2.124^NS^	0.065^NS^
S×N×U		0.019^NS^	1.889^NS^	2.947^NS^	6.225^*^	0.158^NS^	0.156^NS^	0.614^NS^

Values are average mean ± *SE* of five replicates (*n* = 5). Values followed by different letters with the same font-style (non-italic and italic) in the column indicated statistical significance at the *P* < 0.05 level among different treatments according to Tukey’s test. *F*-ratios and levels of significance of three-way ANOVA test: ^NS^Level of significance : not significant. ^*^P < 0.05; ^**^P < 0.01; ^***^P < 0.001.
