# Supplementary material for: Moderate salt treatment alleviates ultraviolet-B radiation caused impairment in poplar plants
Source: Sci Rep. 2016 Sep 6;6:32890. doi: 10.1038/srep32890 (PMC5011775; doi:10.1038/srep32890)
Supplement: Supplementary Information [file srep32890-s1.pdf]

Title: Moderate salt treatment alleviates ultraviolet-B radiation caused impairment in poplar plants

Xuan Ma[1], Yong-Bin Ou[1], Yong-Feng Gao[1], Stanley Lutts[4], Tao-Tao Li[2,3], Yang Wang[1], Yong-Fu Chen[1], Yu-Fang Sun[2,3] & Yin-An Yao[1, 2*]

[1] School of Life Science and Engineering, Southwest University of Science and Technology, Mianyang 621010, China

[2] Key Laboratory of Biogeography and Bioresources, Xinjiang Institute of Ecology and Geography, Chinese Academy of Science, Urumqi 830011, China

[3] University of Chinese Academy of Sciences, Beijing 100039, China

[4] Groupe de Recherche en Physiologie végétale (GRPV); Earth and Life Institute-Agronomy (ELI-A); Université catholique de Louvain, 1348 Louvain-la-Neuve, Belgium

* **Corresponding Author:** Yin-An Yao

School of life science and engineering, Southwest University of Science and Technology, Mianyang 621010, China

E-mail: yaoya@ms.xjb.ac.cn Fax: 86-816-6089534; Tel: 86-991-7823164

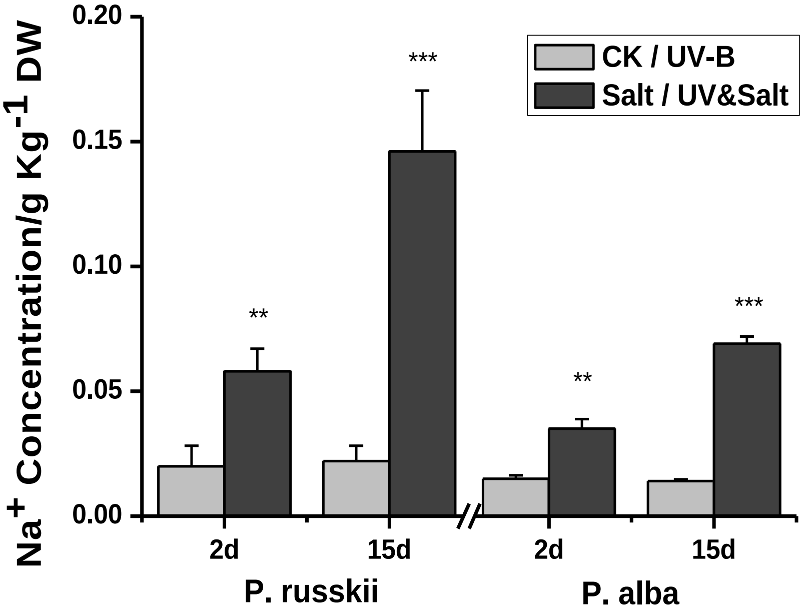

Figure S1. The Na^+ concentration of control (CK, NaCl 0mM), salinity stress (Salt, NaCl 100mM), ultraviolet-B radiation (UV-B) and their combined treatments in leaves of two *Populus* (*P. alba* and *P. russkii*). Date shown are the average mean ± *SE* of six replicates (n = 6). Different numbers of asterisk indicated statistical significance at the $P < 0.05$ level among different treatments according to Turkey's test, ** $P < 0.01$; *** $P < 0.001$.

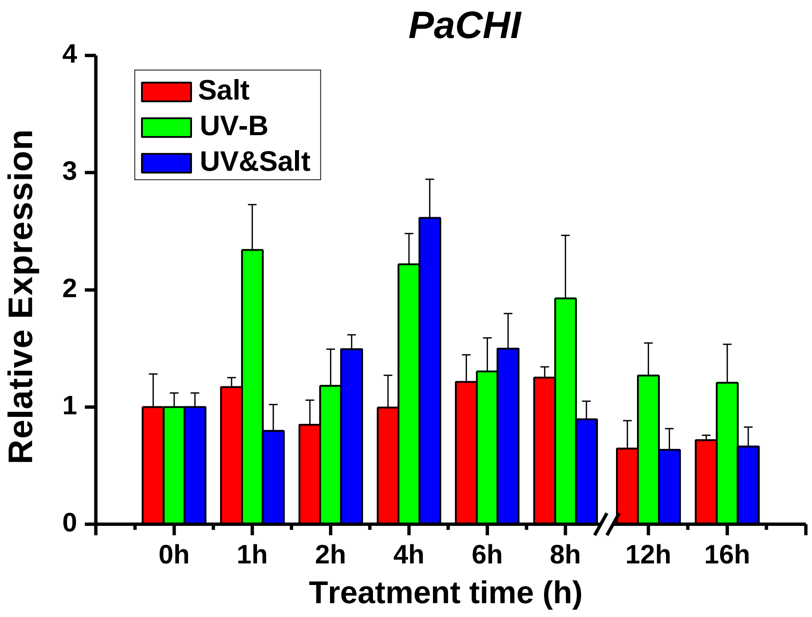

Figure S2. The transcript abundance of *PaCHI* in *P. alba* under UV-B radiation, salt and UV-B &Salt treatments for different treatment duration. 0h, 1h, 2h, 4h, 6h and 8h means the UV-B radiation and salt treatment duration was 0h, 1h, 2h, 4h, 6h and 8h respectively, 12h and 16h means 8h treatment duration (UV-B) in the first day plus the another 4h and 8h treatment duration separately in the second day. The transcript abundance in the control treatment (0h) was set at 1. Data shown are the average mean ± *SE* of three replicates (*n* = 3).

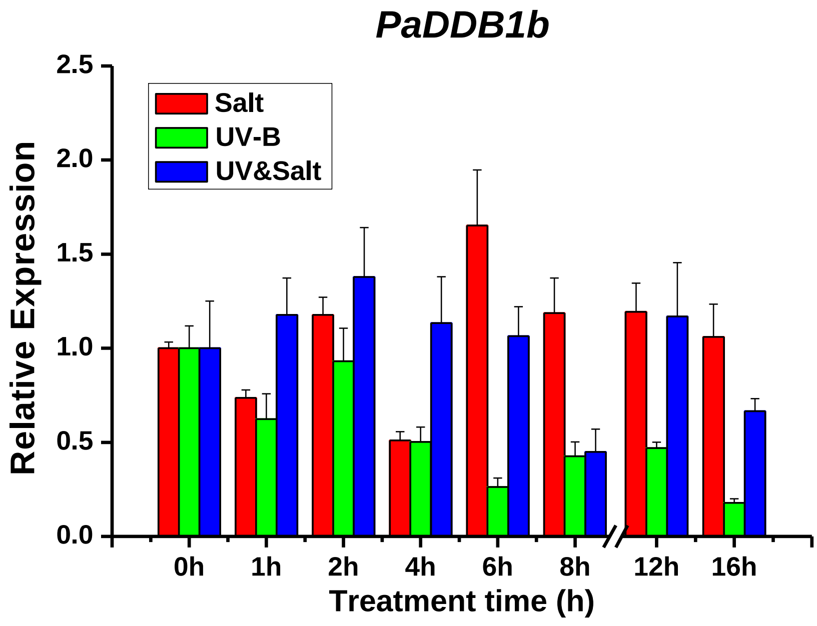

Figure S3. The transcript abundance of *PaDDB1b* in *P. alba* under UV-B radiation, salt and UV-B &Salt treatments for different treatment duration. 0h, 1h, 2h, 4h, 6h and 8h means the UV-B radiation and salt treatment duration was 0h, 1h, 2h, 4h, 6h and 8h respectively, 12h and 16h means 8h treatment duration (UV-B) in the first day plus the another 4h and 8h treatment duration separately in the second day. The transcript abundance in the control treatment (0h) was set at 1. Data shown are the average mean ± *SE* of three replicates (n = 3).

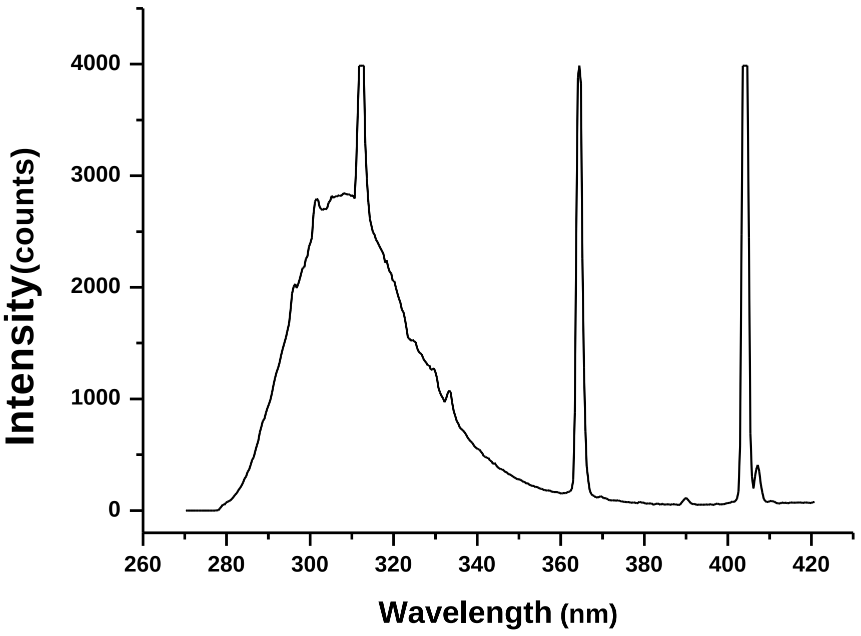

Figure S4. The spectral scan of the ultraviolet fluorescent lamps which were used in our study.

Table S1. Gene description and oligonucleotide primer sequences used in real-time qPCR analysis in *Populus alba*. The primers used in this study were designed by the Primer5 program**,** and synthesized from Sangon Biotech (Sangon Biotech (Shanghai) Co., Ltd., China; http://www.sangon.com/index.jsp).

**Gene abbreviation**	***P. trichocarpa* gene locus**	***Arabidopsis* ortholog locus**	**Tentative annotation**	**Primer Sequences** (forward/reverse(5'—3'))	**Product length(**bp**)**
**PaHY5**	POPTR_0010s00680g	AT5G11260	HY5 family protein	GCGTCAACAGACAGCACATTA/GGAACCAAATCTATGCCAAGTC	188
**PaCOP1**	POPTR_0014s15740g	AT2G32950	photoregulatory zinc-finger protein COP1	TAAGGAACAGGGTCTCAGCC/GCTTTCATCAACCTTAGGCC	201
**PaSTO**	POPTR_0017s05700g	AT1G06040	Salt tolerance-like family protein	CAGTTGATGACTTGCTACAGTTTTC/GGTTGGTGGTGAGATTGGC	173
**PaSTH2**	POPTR_0002s02970g	AT1G75540	Zinc finger protein CONSTANS-LIKE 6	ACAGAAGTAAACAAGGGTGGG/ATCCATAAACGGCAATAGACC	186
**PaFLS1**	POPTR_0019s01130g	AT5G08640	Flavonol synthase 1 protein	TCACCATTCTTGTGCCTAACG/TCCTTGCCTTATCTTTAGCCAC	183
**PaFLS2**	POPTR_0004s14680g	AT5G63590	Flavonol synthase 2 protein	CTATCTGCCATCACCATTCTTG/TTCTTGTCCTATCTTTAGCCACC	193
**PaCHS1**	POPTR_0014s14200g	AT5G13930	chalcone synthase protein	GAAAAGCCTGGTTGAAGCAT/TGCCATAATCACTCAGGACG	167
**PaCHS4**	POPTR_0003s17530g	AT5G13930	naregenin-chalcone synthase protein	CAAACTATTCTACCCAACAGCG/AGAGTTCCAGTCCGAGATGC	162
**PaCHI**	POPTR_0010s21980g	AT3G55120	chalcone isomerase	CCTGGCTCCTCCATTCTTTTC/CAGGAGACACACCGTGCTTG	163
**PaDDB1a**	POPTR_0011s09410g	AT4G05420	Damaged DNA-binding protein 1A	TTTGAGGTTGGTAATGGTGTAATC/ACTATGGCTGAGATCAAGGAATG	117
**PaDDB1b**	POPTR_0001s36830g	AT4G21100	Damaged DNA-binding protein 1B	CTTGAGGTGGTCGGTGAGTATC/AGGTATTGCTCGTGAGGAAGTG	170
**PaDDB2**	POPTR_0001s25810g	AT5G58760	Damaged DNA-binding protein 2	GATATTTTGGCATCTGGCAGT/CCATCATCTTCAGAATCATCATC	170
**PaMSH2**	POPTR_0012s05670g	AT3G18524	muts homolog 2 family protein	TGTCACCCTTGCCCGAGA/ CCTGCTTCAAGTCCATTGTGTC	203
**PaMSH6**	POPTR_0014s11690g	AT4G02070	DNA mismatch repair protein MSH6-1	TGCCCTAAGAGTTATGGTGTCAAT/GGATTAATACTGCCATCTTGTCACA	172
**PaPHR**	POPTR_0001s08030g	AT1G12370	CPD photolyase family protein	GTTGGAAAGAGCGACCGATT/CTGGGGAGCTCCTTCATTGT	169
**Pa-atpI**	Poptr_cp008	ArthCp010	ATP synthase CF0 A subunit	ATTTCACAAAACCTTTATCACTTAGC/TTCACCTATATAAGCCGCAGC	188
**Pa-rbcL**	Poptr_cp030	ArthCp030	Rubisco large subunit	TGTTGATTTACTGCGTGATGATT/CCAAGGGTGCCCTAAAGTTC	199
**PaEF1-α**	POPTR_0006s13310g	AT1G07940	Elongation factor-1 alpha	TGACTGTCACACCTGCCACAT/CAAGAGGAGGATAGGCAGAAAAG	182

Table S2. *F*-ratios and levels of significance of three-way ANOVA test for different physiological and biochemical parameters of two poplar plants (*Populus alba* and *Populus russkii*) under UV-B radiation, salinity stress and their combined treatments. ^{NS}, not significant. $^{*}P< 0.05$; $^{**}P< 0.01$; $^{***}P< 0.001$.

Parameters	Species	Salt	UV-B	Species×Salt	Species×UV-B	UV-B×Salt	Species×UV-B×Salt
**MDA**	1.112^{NS}	0.497^{NS}	3.956^{NS}	1.418^{NS}	0.499^{NS}	0.027^{NS}	1.311^{NS}
**H_2O_2**	2.148^{NS}	0.327^{NS}	12.777^{**}	0.006^{NS}	1.794^{NS}	13.553^{**}	1.556^{NS}
**Proline**	30.524^{***}	83.337^{***}	17.77^{**}	0.058^{NS}	0.094^{NS}	1.354^{NS}	0.762^{NS}
**Glycine betaine**	1517.144^{***}	725.122^{***}	16.245^{**}	422.206^{***}	0.017^{NS}	95.585^{***}	128.126^{***}
**SOD activity**	1876.425^{***}	0.195^{***}	5217.65^{***}	34.043^{***}	522.921^{***}	0.274^{NS}	86.444^{NS}
**POD activity**	6.377^{*}	5.370^{*}	33.973^{***}	2.744^{NS}	0.864^{NS}	1.627^{NS}	2.761^{NS}
**CAT activity**	166.587^{***}	3.094^{NS}	191.381^{***}	10.294^{**}	1.029^{NS}	176.696^{***}	1.824^{NS}
**APX activity**	137.972^{***}	382.796^{***}	137.356^{***}	213.255^{***}	143.546^{***}	260.995^{***}	27.407^{***}
**GR activity**	1656.008^{***}	1334.086^{***}	2235.887^{***}	72.313^{***}	475.395^{***}	514.668^{***}	753.67^{***}
**Total flavonol compounds**	570.262^{***}	28.898^{***}	242.841^{***}	2.542^{NS}	0.203^{NS}	119.817^{***}	14.606^{**}
**Anthocyanins**	23.254^{***}	18.933^{***}	79.653^{***}	39.665^{***}	28.795^{***}	5.446^{*}	0.528^{NS}

Total flavonol compounds showed the total value of rutin, myricetin, quercetin and kaempferol.
